# Association between caffeine intake and constipation in a U.S. population aged 20 to 65 years: Results from NHANES 2007 to 2010

**DOI:** 10.1097/MD.0000000000046853

**Published:** 2026-01-02

**Authors:** Haokang Li, Huanhuan Wang

**Affiliations:** a Guangzhou University of Chinese Medicine, Guangzhou City, Guangdong Province, China; b Nanjing University of Chinese Medicine, Nanjing City, Jiangsu Province, China.

**Keywords:** caffeine, constipation, cross-sectional study, NHANES

## Abstract

Caffeine’s relationship with constipation is unclear. Some studies suggest caffeine may promote bowel movement, while excessive intake could worsen symptoms. This cross-sectional study aimed to evaluate the association between caffeine intake and constipation among U.S. adults aged 20 to 65 years using data from the 2007 to 2010 National Health and Nutrition Examination Survey. Caffeine intake was categorized into quartiles based on 24-hour dietary recall data, and constipation was defined using stool form, bowel movement frequency, and self-reported symptoms. Weighted multivariable logistic regression, restricted cubic spline modeling, and subgroup and interaction analyses were conducted to assess associations and test the robustness of results. Compared with participants in the lowest quartile of caffeine intake (≤35.5 mg/d), those in the third quartile (109.1–228.5 mg/d) had the lowest odds of constipation (odds ratio [OR]: 0.60; 95% confidence interval: 0.44–0.82; *P* =.004). When modeled on a log_2_ scale, each doubling of caffeine intake was associated with a modest reduction in the odds of constipation (OR: 0.95, 95% confidence interval: 0.91–0.99; *P* =.019). Restricted cubic spline analyses supported a nonlinear association (*P* for nonlinear <.01); the curve crossed the null (OR = 1) at approximately 580 mg/d, with no further benefit observed at higher intakes. No significant effect modification was detected across prespecified subgroups. Moderate caffeine consumption was associated with a lower likelihood of constipation, whereas higher caffeine intake did not confer additional benefits. These findings suggest a potential threshold effect of caffeine on bowel function, beyond which further intake may not provide added advantage. Further longitudinal and mechanistic studies are warranted to confirm these associations and clarify the biological pathways linking caffeine consumption to bowel motility.

## 1. Introduction

Chronic constipation, one of the most common functional bowel disorders, affects approximately 8% of adults in the United States according to Rome IV criteria, with significantly higher prevalence among women, and is associated with marked reductions in quality of life and increased gastrointestinal healthcare utilization.^[1]^ The etiology of constipation is multifactorial, with contributions from dietary habits, lifestyle choices, psychological health, and socioeconomic determinants.^[2]^ Consequently, modifications to diet and lifestyle are considered foundational strategies for its prevention and management.^[3]^ Caffeine, a methylxanthine alkaloid and the most widely consumed psychoactive substance globally, is known to exert various physiological effects, including on the gastrointestinal system.^[4]^ However, the association between caffeine intake and constipation remains equivocal, with extant literature presenting conflicting findings. Some evidence suggests a protective association, potentially mediated by caffeine’s prokinetic effects on colonic motor activity and its capacity to favorably modulate the gut microbiota.^[5]^ Conversely, other studies indicate that excessive caffeine consumption could potentially exacerbate constipation or contribute to symptoms of irritable bowel syndrome, possibly by inhibiting intestinal motility in certain contexts.^[6]^The divergent findings in the literature suggest the possibility of a nonlinear, dose-dependent relationship between caffeine intake and the odds of constipation.^[7]^ Therefore, this study aims to investigate the association between caffeine consumption and constipation in the general population.

## 2. Methods

### 2.1. Survey description

National Health and Nutrition Examination Survey (NHANES) is an ongoing, population-based, cross-sectional study. Its purpose is to assess the health and nutritional status of both adults and children in the United States. The survey uses a complex, stratified, multistage probability sampling design to ensure that its findings are representative of the U.S. population. All participants provided written informed consent, and the survey protocol was approved by the Ethics Review Board of the National Center for Health Statistics.

### 2.2. Study population

**Figure 1.**
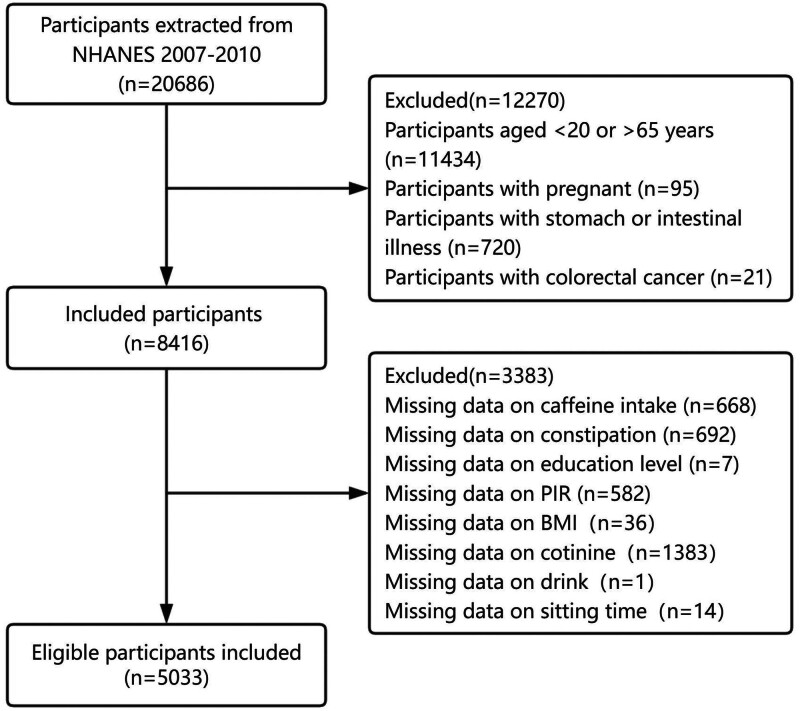
NHANES 2007–2010 participant selection flowchart. NHANES = National Health and Nutrition Examination Survey.

Figure 1 illustrates the screening process used to identify the eligible participants for the study. The study selected participants from the 2007 to 2010 NHANES database because these cycles collected data on both bowel health and sedentary time. Individuals were excluded if they were pregnant, had a self-reported history of colorectal cancer, or had a history of stomach or intestinal illness. The study focused on individuals aged 20 to 65 years, resulting in an initial sample of 8416 participants. Further exclusions were made for those with missing data on caffeine intake, bowel health, or relevant covariates such as education level, poverty income ratio (PIR), body mass index (BMI), serum cotinine, alcohol consumption, and sedentary behavior. After these exclusions, 5033 participants remained in the final analysis.

### 2.3. Caffeine intake

Caffeine intake was assessed in participants of NHANES 2007 to 2010 using two 24-hour dietary recall interviews, including data from the total nutrient intakes and total dietary supplements. The first recall was conducted in person at the mobile examination center, and the second was obtained by telephone 3 to 10 days later. Nutrient and food component totals for each recall were calculated with the USDA Food and Nutrient Database for Dietary Studies (version 4.1), which includes approximately 50 coffee beverages, 30 tea varieties, and both caffeinated and non-caffeinated soft drinks, as well as energy drinks. Each participant’s mean daily caffeine intake was computed by averaging the 2 recall days; when 1 recall was missing, the available day was used. Caffeine intake was then categorized into quartiles: Q1, ≤35.5 mg/d; Q2, 35.6–109.0 mg/d; Q3, 109.1–228.5 mg/d; and Q4, ≥228.6 mg/d. Detailed documentation of the NHANES dietary data structure and calculation procedures is publicly available (https://wwwn.cdc.gov/Nchs/Data/Nhanes/Public/2007/DataFiles/DR1TOT_E.htm).

### 2.4. Constipation definition

Constipation was defined according to the NHANES database as having fewer than 3 bowel movements per week, selecting Type 1 (separate hard lumps, like nuts) or Type 2 (sausage-like but lumpy) on the Bristol Stool Form Scale, or self-reporting being constipated as “always” or “most of the time” over the past year.

### 2.5. Covariates

Covariates included sex, age, race, educational attainment, PIR, BMI, serum cotinine, alcohol consumption, physical activity, sedentary behavior, depressive symptoms, and daily intake of carbohydrates, fiber, fat, total energy, protein, and water. PIR was dichotomized as < 2 or ≥ 2, and BMI was categorized as < 30 kg/m^2^ or ≥ 30 kg/m^2^. Alcohol consumption was classified as Yes/No, based on whether participants reported consuming at least 12 alcoholic beverages in the past year. Physical activity is categorized as light, moderate, or vigorous based on whether the participant participates in moderate or vigorous recreational activities. Sedentary behavior was categorized as Yes/No, with sedentary status defined as ≥ 6 hours of daily sitting time. Depressive symptoms were defined as a score >10 on the Patient Health Questionnaire-9, and categorized as Yes/No.

### 2.6. Statistical analysis

Caffeine intake was modeled both in quartiles and as a continuous variable after log₂ transformation; the corresponding odds ratios quantify the change in the odds of constipation per doubling of daily caffeine intake. Continuous variables (age; serum cotinine; and daily intakes of carbohydrate, fiber, fat, energy, protein, and water) are presented as survey-weighted means ± standard deviations. Categorical variables (sex, race, education level, PIR, BMI, alcohol consumption, physical activity, sedentary behavior, and depression status) are summarized as survey-weighted frequencies and percentages. Between-group comparisons used survey-weighted Student *t* tests for continuous variables and survey-weighted chi-square (χ^2^) tests for categorical variables.

The association between caffeine intake and constipation was assessed using weighted logistic regression models to account for the complex, multistage sampling design of NHANES. Three hierarchical models were constructed. Model 1 was unadjusted. Model 2 adjusted for demographic and lifestyle variables, including sex, age, education level, race, PIR, BMI, serum cotinine, alcohol consumption, physical activity, sedentary behavior, and depression. Model 3 was further adjusted for dietary factors, including carbohydrate, fiber, fat, calorie, protein, and water intake. To assess potential nonlinear associations, restricted cubic spline (RCS) regression models with 3 knots were applied to evaluate the dose–response relationship between caffeine intake and the risk of constipation.

Subgroup analyses and interaction tests were conducted according to demographic characteristics to explore potential effect modification. All statistical analyses were performed using R software (version 4.5.1; R Foundation for Statistical Computing, Vienna, Austria; http://www.r-project.org). Statistical significance was defined as a 2-sided *P*-value < .05.

## 3. Results

### 3.1. Baseline characteristics

Table 1 displays the weighted baseline characteristics of participants, stratified by constipation status. Among the 5033 eligible participants, 607 met the criteria for constipation. The average caffeine intake was 171.55 mg in the non-constipated group and 152.46 mg in the constipated group. Participants were divided into quartiles based on caffeine intake: Q1 (n = 1266), Q2 (n = 1261), Q3 (n = 1250), and Q4 (n = 1256). Significant differences in baseline demographic, lifestyle, and dietary characteristics were observed between participants with and without constipation. Constipation was strongly associated with several demographic factors, lifestyle behaviors, nutritional intake, and depression. Compared to the non-constipated group, those who were female, less educated, had lower income, physically inactive, suffered from depression, and had lower intake of carbohydrates, fiber, fat, calories, protein, and water were more likely to experience constipation. (*P* < .05).

**Table 1:** Weighted characteristics of participants with and without constipation.

Variable	No constipation (n = 4426)	Constipation (n = 607)	*P* value
Age, years, n (%)	41.45 ± 12.85	39.48 ± 13.15	<.001
Sex, n (%)			
Male	2483 (57.0%)	197 (29.7%)	<.001
Female	1943 (43.0%)	410 (70.3%)	
Race, n (%)			
Mexican American	772 (8.1%)	99 (8.6%)	.003
Other Hispanic	426 (4.4%)	79 (6.7%)	
Non-Hispanic White	2092 (69.4%)	253 (62.6%)	
Non-Hispanic Black	918 (11.6%)	152 (16.5%)	
Other	218 (6.5%)	24 (5.5%)	
Education level, n (%)			
Below high school	1181 (18.5%)	200 (24.8%)	.005
High school or above	3245 (81.5%)	407 (75.2%)	
PIR, n (%)			
<2	2176 (34.3%)	367 (45.3%)	<.001
≥2	2250 (65.7%)	240 (54.7%)	
BMI (kg/m^2^), n (%)			
<30	2719 (64.3%)	402 (70.4%)	.039
≥30	1707 (35.7%)	205 (29.6%)	
Cotinine (ng/mL)	84.09 ± 149.00	78.54 ± 137.17	.495
Drink, n (%)			
No	994 (19.2%)	184 (26.7%)	.003
Yes	3432 (80.8%)	423 (73.3%)	
Physical activity, n (%)			
Light	2262 (44.7%)	358 (53.5%)	.002
Moderate	1093 (27.4%)	132 (25.5%)	
Vigorous	1071 (27.9%)	117 (21.0%)	
Sedentary, n (%)			
No	2752 (57.4%)	368 (55.5%)	.525
Yes	1674 (42.6%)	239 (44.5%)	
Depression, n (%)			
No	4032 (93.1%)	492 (82.8%)	<.001
Yes	394 (6.9%)	115 (17.2%)	
Carbohydrate (g/d)	273.85 ± 122.69	245.32 ± 99.22	<.001
Fiber (g/d)	16.94 ± 9.00	13.36 ± 6.85	<.001
Fat (g/d)	86.69 ± 44.12	74.21 ± 37.49	<.001
Calorie (kcal/d)	2294.00 ± 952.71	1978.25 ± 758.98	<.001
Protein (g/d)	89.32 ± 39.73	74.89 ± 33.18	<.001
Water (g/d)	1895.68 ± 1860.48	1442.51 ± 1585.30	<.001
Caffeine intake quartiles (mg/d), n (%)			
Q1 (≤35.5)	1075 (20.0%)	191 (28.4%)	.004
Q2 (35.6–109.0)	1112 (22.8%)	149 (22.5%)	
Q3 (109.1–228.5)	1115 (26.4%)	135 (21.0%)	
Q4 (≥228.6)	1124 (30.9%)	132 (28.0%)	

Continuous variables are presented as weighted means ± standard deviations. Categorical variables are presented as unweighted counts and weighted percentages.

BMI = body mass index, PIR = poverty income ratio.

### 3.2. Relationship between caffeine intake and constipation

Table 2 presents the findings from the weighted multivariable logistic regression analysis. Log2-transformed caffeine intake (log_2_Caffeine) was significantly associated with lower odds of constipation across all models (Model 3: odds ratio [OR]: 0.95, 95% confidence interval : 0.91–0.99; *P* = .019). Compared to the lowest quartile, Q2 (35.6–109.0 mg/d) and Q3 (109.1–228.5 mg/d) showed significantly lower odds of constipation, with Q3 showing the strongest association (Model 3: OR 0.60, 95% confidence interval: 0.44–0.82, *P* = .004). However, Q4 (≥228.6 mg/d) showed no statistically significant association with constipation in adjusted models (Model 2: *P* = .056; Model 3: *P* = .061).

**Table 2:** Weighted multivariable logistic regression of caffeine intake on constipation.

Caffeine intake	Model 1		Model 2		Model 3	
	OR [95% CI]	*P* value	OR [95% CI]	*P* value	OR [95% CI]	*P* value
Continuous [log_2_ (Caffeine)]	0.94 [0.90, 0.97]	.002	0.96 [0.92, 0.99]	.037	0.95 [0.91, 0.99]	.019
Q1	Ref		Ref		Ref	
Q2	0.69 [0.49, 0.99]	.041	0.73 [0.50, 0.86]	.009	0.68 [0.47, 0.99]	.044
Q3	0.56 [0.41, 0.76]	.001	0.62 [0.46, 0.84]	.004	0.60 [0.44, 0.82]	.004
Q4	0.64 [0.51, 0.81]	<.001	0.78 [0.61, 1.01]	.056	0.73 [0.56, 1.02]	.061

Model 1 is unadjusted, while Model 2 adjusts for gender, age, education level, race, PIR, BMI, serum cotinine, alcohol consumption, physical activity, sedentary behavior, and depression. Model 3 additionally adjusts for daily intake of carbohydrates, fiber, fat, calories, protein, and water.

BMI = body mass index, CI = confidence interval, OR = odds ratio, PIR = poverty income ratio.

### 3.3. Nonlinear association between caffeine intake and constipation

**Figure 2.**
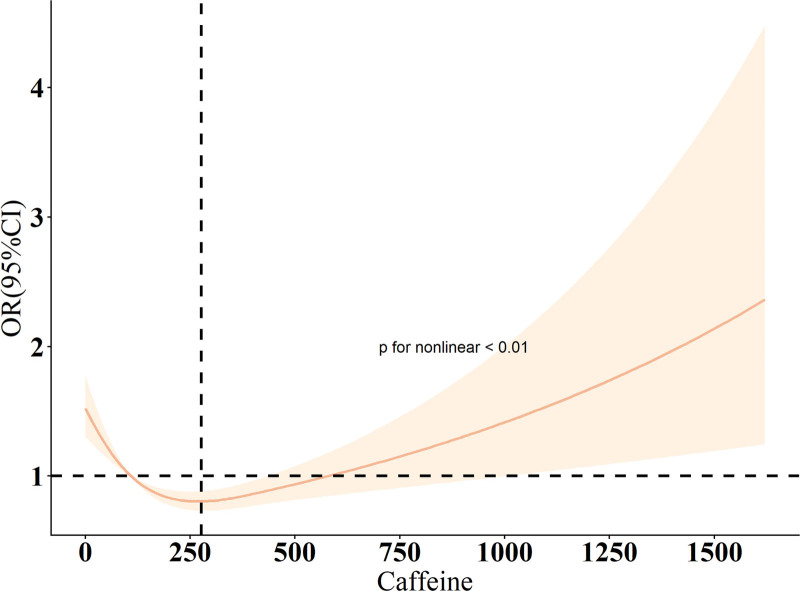
RCS analysis of the association between caffeine intake and constipation. The orange solid line represents the smooth fitted curve between caffeine intake and constipation risk, while the shaded orange area denotes the 95% confidence interval (CI) around the fitted curve. RCS = restricted cubic spline.

RCS analysis revealed a nonlinear association between caffeine intake and constipation (*P* for nonlinear < .01; Fig. 2). The RCS curve presents a U-shaped relationship, with the OR crossing 1 at approximately 109.08 mg/d and 579.76 mg/d.

### 3.4. Subgroup analysis

Table 3 presents the subgroup analyses examining the relationship between caffeine intake and constipation across various demographic and lifestyle characteristics. The inverse association between caffeine intake and constipation remained generally consistent across all examined subgroups, including age, sex, race, educational level, PIR, BMI, alcohol consumption, physical activity and sedentary behavior. No statistically significant interactions were observed between caffeine intake and any of the subgroup variables (*P* for interaction > .05).

**Table 3:** The association between caffeine intake and constipation in different subgroups.

Variable	No constipation	Constipation	*P* for interaction
	OR (95% CI)	OR (95% CI)	
Age, years, n (%)			
<40	Ref	0.96 [0.90, 1.02]	.969
≥40	Ref	0.94 [0.86, 1.01]	
Sex, n (%)			
Male	Ref	0.99 [0.93, 1.06]	.350
Female	Ref	0.93 [0.88, 0.98]	
Race, n (%)			
Mexican American	Ref	1.03 [0.93, 1.13]	.614
Other Hispanic	Ref	0.96 [0.89, 1.04]	
Non-Hispanic White	Ref	0.94 [0.87, 1.00]	
Non-Hispanic Black	Ref	0.97 [0.88, 1.08]	
Other	Ref	0.95 [0.84, 1.08]	
Education level, n (%)			
Below high school	Ref	0.93 [0.88, 0.98]	.264
Above high school	Ref	1.00 [0.93, 1.07]	
PIR, n (%)			
<2	Ref	0.92 [0.87, 0.97]	.402
≥2	Ref	0.97 [0.93, 1.03]	
BMI (kg/m^2^), n (%)			
<30	Ref	0.94 [0.89, 1.00]	.858
≥30	Ref	0.96 [0.87, 1.05]	
Drink, n (%)			
No	Ref	0.97 [0.90, 1.04]	.531
Yes	Ref	0.94 [0.90, 0.98]	
Physical activity, n (%)			
Light	Ref	0.96 [0.89, 1.02]	.999
Moderate	Ref	0.93 [0.85, 1.02]	
Vigorous	Ref	0.94 [0.87, 1.01]	
Sedentary, n (%)			
No	Ref	0.98 [0.92, 1.04]	.295
Yes	Ref	0.90 [0.85, 0.96]	

CI = confidence interval, OR = odds ratio.

## 4. Discussion

In this cross-sectional analysis of U.S. adults aged 20 to 65 years, we observed a statistically significant nonlinear association between caffeine intake and the prevalence of constipation. The lowest odds of constipation were observed among individuals with moderate caffeine intake of approximately 109 to 229 mg/d. Higher intake levels were not associated with a further decrease in OR (*P*>.05). RCS analysis indicated that the risk increased significantly only when intake exceeded approximately 580 mg/d. These findings suggest a potential threshold effect in which moderate caffeine intake is associated with reduced constipation risk, while excessive consumption offers no further benefit and may even be detrimental. Given the cross-sectional design, residual confounding and reverse causation cannot be excluded. Prospective studies and mechanistic investigations are needed to corroborate these associations and clarify the biological basis of the apparent threshold.

Clinical research suggests that the relationship between caffeine and constipation may vary by dose and across different population subgroups. Early physiological experiments found that approximately one-third of healthy individuals report an urge to defecate shortly after drinking coffee; notably, this effect has been observed with both caffeinated and decaffeinated coffee, suggesting that caffeine and other coffee constituents may jointly act on the gut.^[8,9]^ Colonic motility studies have also demonstrated that coffee can enhance contractions of the rectosigmoid colon, thereby promoting defecation.^[10–12]^ Moreover, meta-analyses indicate that postoperative coffee consumption significantly shortens the time to first flatus and defecation, thereby aiding recovery from ileus.^[13,14]^ Collectively, these clinical data support the concept that moderate coffee or caffeine intake may promote gut motility and alleviate constipation. However, several dietary epidemiological studies have found that high energy intake, sugar-sweetened beverages and caffeinated beverage consumption may be associated with worsening constipation symptoms.^[15,16]^

Several potential mechanisms may underpin this nonlinear relationship. First, At low to moderate doses, caffeine may stimulate both sympathetic and parasympathetic pathways, enhancing the gastrocolic reflex and colonic propulsive contractions and thereby shortening intestinal transit time.^[10]^ Caffeine and coffee polyphenols may also modulate the gut microbiota – increasing beneficial taxa and short-chain fatty acid production – which can improve motility.^[17]^ Furthermore, specific coffee components may regulate intestinal smooth muscle contraction and relaxation via effects on calcium channels.^[18]^ However, at higher concentrations, caffeine’s physiological effects may become counterproductive.^[19,20]^ Excessive sympathetic nervous system activation can inhibit gastrointestinal motility.^[4]^ High caffeine intake has also been hypothesized to disrupt microbial homeostasis, potentially favoring the growth of methane-producing organisms that can slow colonic transit.^[21,22]^ These divergent, dose-dependent mechanisms provide a compelling rationale for the threshold effect observed in our analysis. Moreover, inter-individual variations in caffeine metabolism, influenced by genetic polymorphisms and age-related physiological changes, may render some individuals more susceptible to the adverse gastrointestinal effects of high caffeine consumption.^[23,24]^

From a public health perspective, our findings underscore the importance of dose when considering caffeine intake for constipation management. Low to moderate intake may represent a simple, cost-effective dietary adjunct to improve bowel function, whereas excessive consumption offers no additional benefit and may increase risk in certain subgroups. Although our study did not stratify by all potential effect modifiers, prior research suggests that women, individuals with obesity, and patients with irritable bowel syndrome may exhibit heightened sensitivity to caffeine.^[25]^ Accordingly, dietary guidance should account for individual tolerance and population heterogeneity and consider tailored recommendations for caffeine intake. As a practical illustration, a 250 mL cup of black coffee with 30 g of dark chocolate, or two 250 mL cups of red or green tea with 30 g of dark chocolate, corresponds to approximately 109 to 229 mg of caffeine per day. As a non-coffee/tea option, one 500 mL energy drink plus 30 g of dark chocolate provides approximately 184 mg/d (values vary by brand and formulation).

This study has several limitations. First, caffeine intake was estimated based on a 24-hour dietary recall, which may not accurately reflect long-term consumption patterns and is susceptible to recall bias. Second, the cross-sectional design inherently limits our ability to establish a temporal relationship between caffeine intake and constipation, thereby precluding causal inference. Longitudinal cohort studies are necessary to delineate the causal pathway and to more precisely define the intake threshold. Finally, due to limited data access, there may be other confounding factors that were not accounted for. To better control for these confounders, randomized controlled trials are needed.

## 5. Conclusion

In this cross-sectional analysis of a nationally representative sample of U.S. adults, we observed a significant nonlinear, U-shaped association between caffeine intake and constipation. Moderate caffeine consumption (approximately 109–229 mg/d) was linked to the lowest odds of constipation, whereas higher intake levels did not yield additional benefits. These findings suggest a potential role for moderate caffeine intake in constipation management. Future prospective cohort studies and randomized controlled trials are needed to validate these findings and to determine the optimal therapeutic range of caffeine intake across diverse populations.

## Acknowledgments

The authors thank the database staff and participants for their valuable contributions.

## Author contributions

**Conceptualization:** Haokang Li.

**Data curation:** Haokang Li.

**Methodology:** Huanhuan Wang.

**Visualization:** Huanhuan Wang.

**Writing – original draft:** Haokang Li.

**Writing – review & editing:** Huanhuan Wang.
